# Integration of Baseline Metabolic Parameters and Mutational Profiles Predicts Long-Term Response to First-Line Therapy in DLBCL Patients: A Post Hoc Analysis of the SAKK38/07 Study †

**DOI:** 10.3390/cancers14041018

**Published:** 2022-02-17

**Authors:** Sofia Genta, Guido Ghilardi, Luciano Cascione, Darius Juskevicius, Alexandar Tzankov, Sämi Schär, Lisa Milan, Maria Cristina Pirosa, Fabiana Esposito, Teresa Ruberto, Luca Giovanella, Stefanie Hayoz, Christoph Mamot, Stefan Dirnhofer, Emanuele Zucca, Luca Ceriani

**Affiliations:** 1 Clinic of Medical Oncology, Oncology Institute of Southern Switzerland, Ente Ospedaliero Cantonale, 6500 Bellinzona, Switzerland; gentasofia@gmail.com (S.G.); maria.pirosa@eoc.ch (M.C.P.); fabiana.esposito@eoc.ch (F.E.); emanuele.zucca@eoc.ch (E.Z.); 2 Clinic of Hematology, Oncology Institute of Southern Switzerland, Ente Ospedaliero Cantonale, 6500 Bellinzona, Switzerland; guido.ghilardi19@gmail.com; 3 Institute of Oncology Research, Faculty of Biomedical Sciences, Università della Svizzera Italiana, 6500 Bellinzona, Switzerland; luciano.cascione@ior.usi.ch; 4 Swiss Institute of Bioinformatics, 1015 Lausanne, Switzerland; 5 Institute of Medical Genetics and Pathology, University Hospital Basel, University of Basel, 4031 Basel, Switzerland; darius.juskevicius@usb.ch (D.J.); alexandar.tzankov@usb.ch (A.T.); stephan.dirnhofer@usb.ch (S.D.); 6 Swiss Group for Clinical Cancer Research (SAKK) Coordinating Center, 3008 Bern, Switzerland; saemi.schaer@gmail.com (S.S.); stefanie.hayoz@bfh.ch (S.H.); 7 Clinic of Nuclear Medicine and PET/CT Center, Imaging Institute of Southern Switzerland, Ente Ospedaliero Cantonale, 6500 Bellinzona, Switzerland; lisa.milan@eoc.ch (L.M.); teresa.ruberto-macchi@eoc.ch (T.R.); luca.giovanella@eoc.ch (L.G.); 8 Department of Nuclear Medicine, University Hospital Zurich, University of Zurich, 8006 Zurich, Switzerland; 9 Division of Oncology, Cantonal Hospital Aarau, 5001 Aarau, Switzerland; christoph.mamot@ksa.ch; 10 Department of Medical Oncology, Bern University Hospital, University of Bern, 3008 Bern, Switzerland

**Keywords:** PET/CT, mutational profile, DLBCL, lymphoma, prognostic index

## Abstract

**Simple Summary:**

In this manuscript, we present a statistical model for reliable and early prediction of treatment failure in patients with diffuse large B-cell lymphoma. The model combines measurable parameters—namely, the metabolic tumor volume and the metabolic heterogeneity, from baseline PET/CT with the presence or absence of mutations in *SOCS1* and *CREBBP/EP300* and represents a promising tool for the design of clinical trials focused on tailoring treatment to the individual risk. According to our bioinformatics analysis, mutation profiling may not be needed in patients with high-risk PET/CT metrics. Hence, the proposed approach may help optimize economic resources avoiding costly, and likely unnecessary, DNA analysis in many patients.

**Abstract:**

Accurate estimation of the progression risk after first-line therapy represents an unmet clinical need in diffuse large B-cell lymphoma (DLBCL). Baseline (18)F-fluorodeoxyglucose positron emission tomography/computed tomography (PET/CT) parameters, together with genetic analysis of lymphoma cells, could refine the prediction of treatment failure. We evaluated the combined impact of mutation profiling and baseline PET/CT functional parameters on the outcome of DLBCL patients treated with the R-CHOP14 regimen in the SAKK38/07 clinical trial (NCT00544219). The concomitant presence of mutated *SOCS1* with wild-type *CREBBP* and *EP300* defined a group of patients with a favorable prognosis and 2-year progression-free survival (PFS) of 100%. Using an unsupervised recursive partitioning approach, we generated a classification-tree algorithm that predicts treatment outcomes. Patients with elevated metabolic tumor volume (MTV) and high metabolic heterogeneity (MH) (15%) had the highest risk of relapse. Patients with low MTV and favorable mutational profile (9%) had the lowest risk, while the remaining patients constituted the intermediate-risk group (76%). The resulting model stratified patients among three groups with 2-year PFS of 100%, 82%, and 42%, respectively (*p* < 0.001).

## 1. Introduction

Diffuse large B-cell lymphoma, not otherwise specified (DLBCL, NOS), is the most frequent aggressive lymphoid malignancy in adult patients, accounting for nearly 30% of non-Hodgkin lymphomas (NHL) [1]. The addition of the anti-CD20 monoclonal antibody rituximab (R) to standard first-line chemotherapy with cyclophosphamide, doxorubicin, vincristine, and prednisone (CHOP) has improved patient survival [2]. However, about 35–40% of patients either fail to respond to this treatment or, after a temporary remission, eventually relapse [3]. Therapeutic opportunities for patients with refractory or recurrent disease are limited and their outcome depends on many factors including the response to first-line treatment, the length of the remission, and the possibility to receive high-dose chemotherapy supported by autologous stem cell transplant (ASCT). Overall, less than one-third of patients are alive 4 years after relapse [4,5]. Nowadays, additional promising treatments, including chimeric antigen receptor (CAR) T-cell therapy, polatuzumab vedotin, and T-cell-engaging bispecific antibodies, have been developed for chemotherapy-resistant lymphomas, allowing cures even in highly pretreated patients [6,7,8,9,10,11]. Thus, early recognition of the high-risk patients destined to relapse after first-line treatment could optimize the therapeutic strategies.

In the past few decades, great efforts have been made to identify reliable prognostic markers. Using a score based on the age of the subjects, the stage of the disease, the number of extra-nodal sites, the performance status, and the LDH level, the International Prognostic Index (IPI) and its updated versions—revised IPI (R-IPI) and NCCN enhanced-IPI—allow the separation of patient subsets with different prognosis [12,13,14]. Their easy availability makes these indices widely used [15]. However, the incorporation of further information such as molecular and imaging features might result in higher predictive power [16,17]. The metabolic response in 18F-fluorodeoxyglucose (18FDG) positron emission tomography/computed tomography (PET/CT) scan at the end of treatment is currently the best predictor of patient survival [18]. Increasing evidence suggests that baseline PET/CT parameters, including metabolic tumor volume (MTV), total lesion glycolysis (TLG) [19,20,21,22,23], and metabolic heterogeneity (MH) and radiomic features indicating lymphoma dissemination, represent independent and powerful prognostic factors in DLBCL [24,25,26].

Modern molecular analyses have identified multiple genetic factors that could help predict the outcome of DLBCL patients [27,28,29,30,31], and it has been suggested that the combined evaluation of quantitative PET parameters with biological and genetic features may allow a more refined risk stratification [32,33].

We already developed prognostic models based on functional parameters from PET/CT scans [24,25] of DLBCL patients homogeneously treated with the R-CHOP14 regimen in the SAKK38/07 clinical trial [34]. In the same cohort, phenotypic and genotypic studies identified several potential biomarkers [35,36], with *CREBBP* and *EP300* mutations being strongly associated with worse outcomes at multivariable analysis [36]. The present manuscript reports additional post hoc studies investigating whether the integration of mutation profiling and PET metrics may further improve the identification of patients at high risk of relapse, who could potentially benefit from an early intensification of treatment (e.g., more aggressive induction regimens or autologous stem cell consolidation).

## 2. Patients and Methods

This study was approved by the Institutional Review Board/Ethics Committee of the participating centers and was conducted in accordance with the ethical standards of the 1964 Declaration of Helsinki and its later amendments.

### 2.1. Study Population

In a prospective study known as the SAKK 38/07 trial of the Swiss Group for Clinical Cancer Research, 156 patients with a diagnosis of DLBCL were treated with 6 cycles of R-CHOP (rituximab 375 mg/m^2^, cyclophosphamide 750 mg/m^2^, doxorubicin 50 mg/m^2^, vincristine 1.4 mg/m^2^ on day 1, and prednisone 100 mg/m^2^ for 5 days) repeated at 14-day intervals, followed by 2 cycles of rituximab (375 mg/m^2^). Consolidation radiotherapy was allowed, if clinically indicated, and was administered in 21 patients (15%), in accordance with local guidelines [34]. The diagnosis of DLBCL was confirmed by central pathology review in all cases, and the presence of “double-hit” cases with *BCL2* and *C-MYC* rearrangements was excluded by in situ hybridization analysis [35]. We estimated the patients’ risk according to the IPI [12] and its main variants—R-IPI [13] and NCCN enhanced-IPI [14]. The cell-of-origin (COO) was centrally determined by immunohistochemistry using the Hans algorithm [37].

### 2.2. Mutational Profile Evaluation

Tumor specimens for molecular analysis were available for 72 patients. As detailed elsewhere [36], somatic mutations were detected by targeted high-throughput sequencing (HTS) using a target enrichment panel covering either mutational hotspots or all exons of 68 genes most frequently mutated in B-cell lymphomas. Sequencing was performed by the Ion Torrent S5 XL machine (Thermo Fisher Scientific, Carlsbad, CA, USA).

### 2.3. PET/CT Images Analysis

All patients performed PET/CT scans at diagnosis, after 2 cycles of R-CHOP14, and at the end of immunochemotherapy. All PET/CT scans were centrally reviewed using a standard protocol with dedicated imaging software (MM Oncology, Syngo.via, Siemens). For MTV estimation, the tumor lesions were segmented with a fixed threshold at a standardized uptake value (SUV) of 2.5. Maximum SUV (SUVmax) and TLG were then calculated automatically. MH of the target lesion (i.e., the lesion with the highest 18FDG uptake) was measured in each patient by the method of the area under the curve of cumulative SUV-volume histogram (AUC-SH) [38]. In a prior study of the same patient population, we established the optimal cut-off values of functional PET/CT parameters using receiver-operating characteristic (ROC) analysis [24]. In particular, the MTV cut-off point was 931 mL for progression-free survival (PFS) and 1149 mL for overall survival (OS), respectively, while the MH cut-off point was 0.43 AUC-CSH for both PFS and OS.

### 2.4. Statistical Analysis

Continuous variables are shown as medians and interquartile ranges (IQR) and categorical variables as percentages. Continuous or ordinal variables were compared using Student’s *t*-test. The chi-square test or Fisher exact test were used for categorical variables, as appropriate. PFS was calculated from treatment start to progression or death from any cause, OS from treatment start to death from any cause. Survival curves were generated using the Kaplan–Meier method and compared using the log-rank test (or the log-rank test for trend, as appropriate). Multivariable analysis and estimation of hazard ratio (HR) were conducted using Cox proportional hazard models.

We built the classification tree using the unsupervised recursive-partitioning method to develop unbiased models implemented into the “*ctree*” function of the R package ‘*party*’ [39]. The tested dichotomized variables were the individual components of the IPIs—namely, age (>60 years), LDH (>normal), Ann Arbor stage (>2), extranodal involvement (>1 site), Eastern Cooperative Oncology Group (ECOG) performance status (≥2), MTV (>cut-off value), MH (>cut-off value), and the presence or absence of a favorable mutation profile (*SOCS1* mutated and *CREBBP/EP300* wild type). In each node, the variable that best discriminated the population in analysis (according to PFS or OS, as appropriate) was used to separate the subjects into two branches.

The predictive accuracy of different prognostic models was compared using the Harrell C concordance probability estimate (CPE) [40]. C statistics range from 0.5 to 1, and higher values of C indicate more accurate discrimination. The relative quality of the models was also assessed by the Akaike information criterion (AIC), which estimates the likelihood of a prognostic model to predict future outcomes [41]. The optimal model is the one with minimum AIC (i.e., best fit) in comparison with all the others.

All statistical tests were two sided. Statistical significance was defined by a *p*-value < 0.05. Negative predictive value (NPV) and positive predictive value (PPV) were calculated according to standard definitions. The analysis was performed with the Statistical Package for the Social Sciences software, (SPSS version 22.0, Chicago, IL, USA) or the R statistical software environment (version 3.1.1) as appropriate.

## 3. Results

Clinical characteristics and outcomes of the patients enrolled in the SAKK 38/07 study have been published in a previous report [34]. In total, 141 patients with baseline 18FDG-PET/CT scans suitable for imaging post-processing and a complete clinical follow-up were included in the current analysis. The main characteristics of the study population at baseline are summarized in Table 1. After a median follow-up time of 64 months (IQR 60–67 months), 30 patients experienced disease progression, while 23 patients died.

### 3.1. Mutational Profile Impact on Outcome

In 72 (51%) patients, we found mutations affecting 46 (68%) of the investigated genes [36]. The most frequently mutated gene was *KMT2D*, (lysine methyl-transferase 2D) in approximately 35% of cases, followed by *SOCS1* (suppressor of cytokine signaling 1) in 25%, *ATM* (ataxia telangiectasia mutated serine/threonine kinase) in 19%, GNA13 (G protein subunit alpha 13) in 18%, and *B2M* (beta-2 microglobulin) in 15% (Table 2). Given the close functional interaction between the two acetyltransferase genes, *CREBBP* (CREB binding protein) and *EP300* (E1A binding protein p300) [42], and their combined prognostic value [36], these were evaluated together. *CREBBP*/*EP300* mutations were found in 19% of the cases.

In keeping with our prior study, which included the patients of the present cohort and others with no available PET/CT scans [36], the subjects harboring *CREBBP*/*EP300* mutations had inferior PFS in comparison with those carrying wild-type genes (49% vs. 82% at 2 years, *p* = 0.002). Alterations affecting *CREBBP* or *EP300* were also predictive of shorter OS (79% vs. 93% at 2 years, *p* = 0.001).

The favorable prognostic impact on PFS of *SOCS1* mutations described in the prior report [36] was maintained, although with borderline significance, also in the cohort analyzed by the present study (2-year PFS 93% vs. 70%, *p* = 0.054), while there was no evident impact on OS (100.0% vs. 86.8% at 2 years, *p* = 0.167).

**Figure 1:**
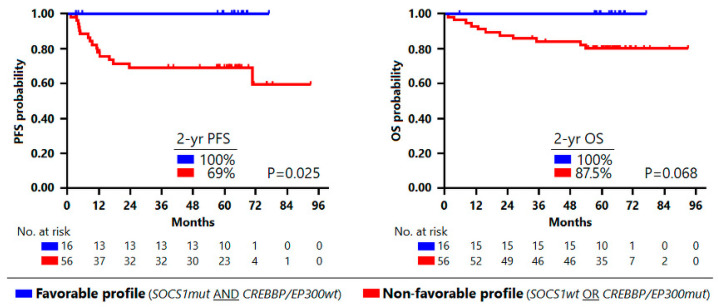
Survival analysis according to mutation profiling. Kaplan–Meier curves estimate progression-free survival (**left**) and overall survival (**right**) by mutational profile. In blue: patients with favorable profile; in red: patients with non-favorable profile.

Based on these results, we categorized two subsets of patients, one with a favorable mutational profile (*n* = 16), defined by the presence of *SOCS1* mutations in the absence of *CREBBP* and *EP300* mutations, and the other, with a non-favorable mutational profile (*n* = 56), characterized by wild-type *SOCS1* and/or mutated *CREBBP/EP300*. Interestingly, none of the patients with a favorable mutational profile relapsed during the entire follow-up period, and their PFS rates were better than in the rest of the patients (100% vs. 69% at 2 years, *p* = 0.025). A similar trend (100% vs. 87.5%) was observed also for OS, albeit this was not statistically significant (Figure 1).

### 3.2. Classification Trees for Outcome Prediction

**Figure 2:**
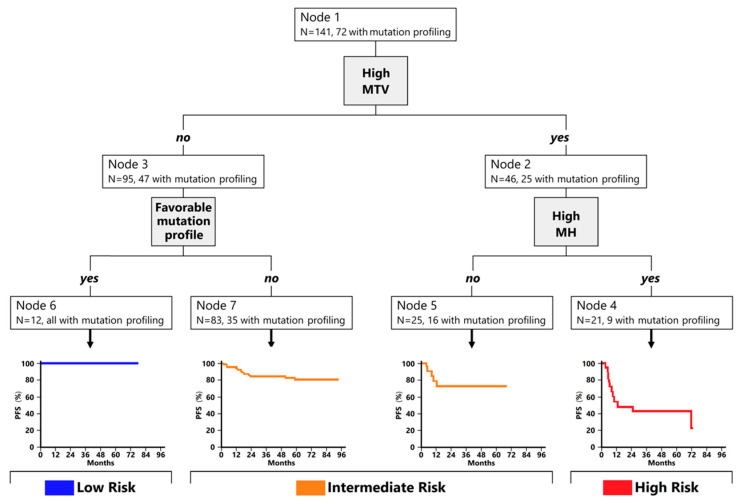
Classification tree analysis for PFS prediction. Node 1: A total of 141 patients were included for the current analysis. Patients with high MTV were sorted in node 2 (N = 46). Node 2 was then sorted based on MH. Patients with MTV and MH high were clustered into node 4 (N = 21), while patients with MTV high and MH low were clustered in node 5 (N = 25). Patients with low MTV (node 3; N = 95) were then evaluated for the presence of a known favorable mutational profile. Patients with favorable mutational profiles and low MTV were classified in node 6 (N = 12). Patients with low MTV and without a known favorable mutational profile constituted node 7 (N = 83). Node 4 was defined as high-risk group, while node 6 was defined as low-risk group. Nodes 5 and 7 were then merged in a single group, referred to as intermediate-risk group.

We then assessed whether the integration of the mutational profile and functional PET/CT parameters could better predict the PFS in first-line treated DLBCL in comparison with clinical prognostic scores. Using an unsupervised recursive partitioning algorithm including the dichotomized clinical prognostic factors that contribute to the IPI, along with imaging parameters (MTV and MH) and genetic features (wild-type *SOCS1* and/or mutated *CREBBP/EP300*), which had a significant impact on the outcome in univariable analysis, we generated a prognostic model that categorized patients among three groups with different outcomes—namely, high-risk group (high MTV and high MH, N = 21), low-risk group (low MTV and favorable mutation profile, N = 12), and intermediate-risk group (all the rest, N = 108) (Figure 2).

**Figure 3:**
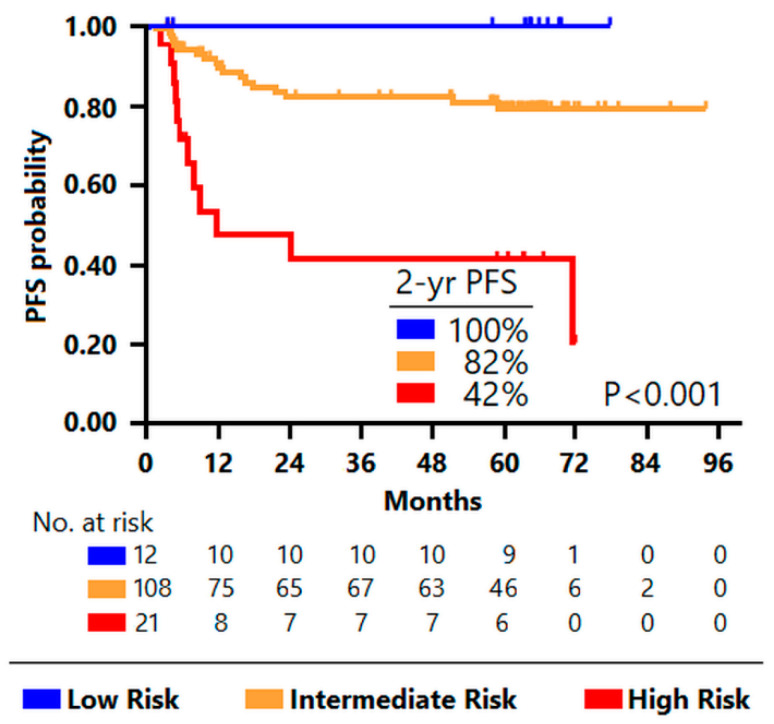
Kaplan–Meier progression-free survival estimates according to the integrative PET model derived from the classification tree. The blue curve shows survival of low-risk patients, the yellow curve, of intermediate-risk patients, and the red one, of high-risk patients. Notably, 89% of the high-risk patients had a non-favorable mutational profile.

The three groups had different 2-year PFS (42% for the high-risk group, 82.0%, for the intermediate-risk group, and 100% for the low-risk group). Most patients in the high-risk group experienced early relapse (median PFS 11.4 months vs. not reached in the other two groups, *p* < 0.001). The algorithm, based on the integration of PET and tumor genotyping results, predicted disease progression or relapse with a PPV of 57% (high-risk group) and an NPV of 100% (low-risk group) (Figure 3).

Patients’ characteristics according to the three risk groups are shown in Table 3. Aside from the expected significantly biased distribution of the mutational profile, the groups did not differ in terms of age, presence of extranodal involvement, performance status, and COO subtype, while the high-risk group predominantly comprised patients with high LDH, advanced stage, and unfavorable IPI.

**Figure 4:**
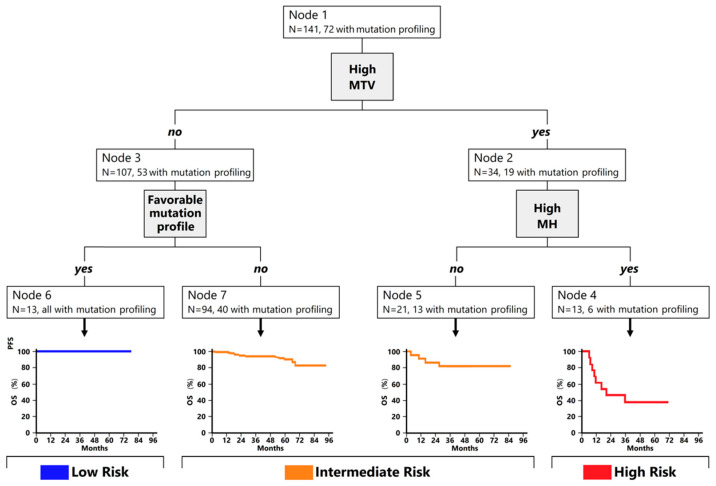
Classification tree analysis for overall survival prediction. Node 1: A total of 141 patients were included for the current analysis. Patients with high MTV were sorted in node 2 (N = 34). Node 2 was then sorted based on MH. Patients with MTV and MH high were clustered into node 4 (N = 13), while patients with MTV high and MH low were clustered in node 5 (N = 21). Patients with low MTV (node 3; N = 107) were then evaluated for the presence of a known favorable mutational profile. Patients with favorable mutational profiles and low MTV were classified in node 6 (N = 13). Patients with low MTV and without a known favorable mutational profile constituted node 7 (N = 94). Node 4 was defined as high-risk group, while node 6 was defined as low-risk group. Nodes 5 and 7 were then merged in a single group, referred to as intermediate-risk group.

Analogous to PFS, the application to OS of the unsupervised recursive partitioning approach identified the same risk groups: high risk for patients with high MTV and high MH (N = 13), low risk for patients with low MTV and favorable mutational profile (N = 13), and intermediate risk for the 115 remaining patients (Figure 4).

**Figure 5:**
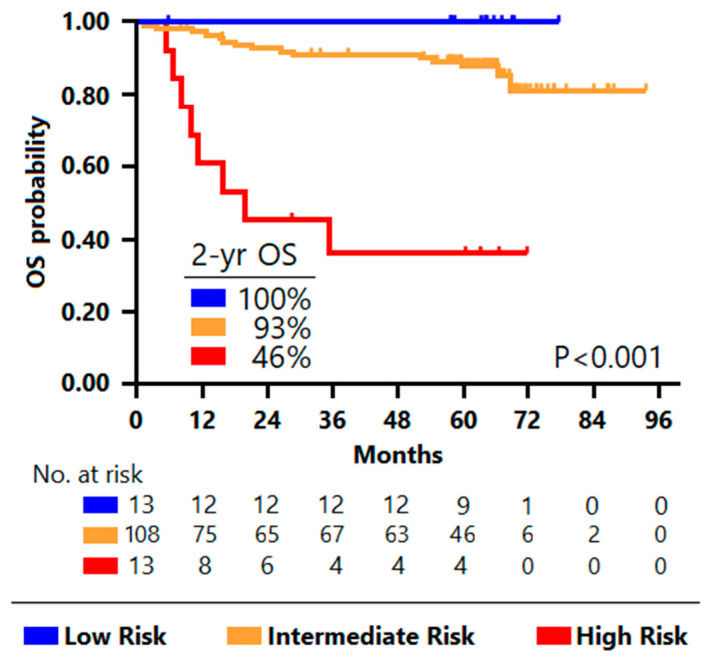
Kaplan–Meier overall survival estimates according to the integrative PET model derived from the classification tree. The blue curve shows the survival of low-risk patients, the yellow curve, of intermediate-risk patients, and the red one, of high-risk patients. Notably, all the high-risk patients had a non-favorable mutational profile.

The 2-year OS was 46% among high-risk patients, 93% in the intermediate-risk group, and 100% in the low-risk group (*p* < 0.001) (Figure 5). Hence, the model derived from the classification tree analysis predicted patient death with PPV of 61.5% (high-risk group) and NV of 100% (low-risk group).

### 3.3. Comparison between Prognostic Models

A concordance probability analysis showed that this model (Harrell C 0.67 for PFS and 0.69 for OS) improved the prognostic capacity of either the MTV (Harrell C 0.64 for PFS and 0.68 for OS) or the mutational profile alone (Harrell C 0.62 for both PFS and OS). In multivariable analysis (Table 4), the above-described model that combines functional imaging and molecular features remained the only independent predictor of PFS and OS after controlling for the most widely used clinical indices—namely, IPI, R-IPI, and NCCN-IPI, which, in a prior study on the same cohort, were significant predictors of OS and had a borderline impact on PFS [24]. The classification tree-derived model was also the only factor significantly affecting PFS and OS in Cox models, including the individual factors contributing to the construction of IPI, revised-IPI, and NCCN-IPI (data not shown).

Table 5 shows the performance of different prognostic models assessed by using either the Akaike information criterion (the lowest value indicates the model that loses less information, i.e., the one with the highest quality) or a concordance probability index, the Harrell C-statistic (the higher C values, the better prediction ability). The discriminatory power and predictive accuracy of our model, which combines functional imaging and molecular features, were superior, for both PFS and OS, to the ones of the International Prognostic Indices (IPI, R-IPI, and NCCN-IPI).

## 4. Discussion

A reliable and solid early estimate of the risk of relapse after initial treatment may tremendously improve the management of DLBCL patients. During the past several years, a variety of clinical, molecular, and imaging features have been shown to predict clinical outcomes after first-line therapy. The best individual prognostic factor is the metabolic response on PET/CT at the end of treatment [18].

The combination of PET/CT-derived quantitative parameters with the genetic profile, in the so-called integrative PET, has been hypothesized to provide a powerful prognostic tool to complement clinical data for treatment decisions [43,44]. Only a few studies have explored this option so far [33,45], their main limitation being the lack of methodology standardization.

Our group identified *CREBBP*/*EP300* mutations as predictors of poor outcomes in patients with newly diagnosed DLBCL treated with R-CHOP, while *SOCS1* mutations were associated with better prognoses [36]. In the current post hoc analysis, we demonstrated that the combination of these mutational features enables the distinction between patients with favorable and non-favorable mutational profiles, with significant impacts on the risk of relapse. We integrated these molecular data with functional PET/CT metrics to generate a PFS prognostic model, which was able to risk-stratify patients and to accurately identify the subsets of patients who will most likely either be cured or progress. Interestingly, the model is entirely based on information available prior to treatment, allowing upfront prediction of patient outcome and keeping its validity for the prediction of OS. Patients in the high-risk subgroup had more frequently elevated LDH and high IPI scores than the other groups. This is not surprising as LDH is a surrogate marker of disease burden: we might speculate that increased LDH levels reflect the elevated MTV, which this analysis confirmed to be an optimal individual parameter to identify patients at high risk of progression or death [17,22,24,25].

Our prior studies already indicated that the concomitant presence of high MTV and MH could reflect a disease with more aggressive behavior, leading to failure of first-line R-CHOP therapy and thus requiring additional treatment [24]. This is confirmed by the present analysis, which indicates that the combined powerful prognostic capacity of MTV and MH is independent of the presence of specific gene mutations. In our model, however, the addition of the mutational profiling data allowed a better risk stratification of patients with low MTV, with an NPP of 100%.

Recent advances in molecular biology have led to the identification of mutational signatures, proposed as useful tools to study DLBCL, even if their prognostic role has so far not been established [27,28,36,46,47]. Nevertheless, the elevated costs of such evaluations could potentially limit the number of patients that can benefit from them. A key point of our model is in fact that only patients without high-risk PET/CT parameters should be candidates to perform prognostic mutational tests, resulting, indeed, in an optimization of the economic resources.

The main limitation of the current retrospective study consists of the small number of patients. Only 72 of the 141 included patients had an evaluable mutational status. Indeed, the study sample size prevented proper cross-validation, and therefore, the hypothesis generated by our results needs to be confirmed by further studies in external patient cohorts. Nonetheless, the unsupervised recursive-partitioning method adopted to build the classification tree may have partially mitigated the limitations related to the number of patients with missing mutation analysis. In fact, the process considered all patients, without completely losing the information provided by the patients who otherwise would have been excluded.

The limited sample size may also explain why the somatic mutations in several genes, which, in other studies, have been described to be predictive of early progression (e.g., *CARD11, BCL2, BCL6, BCL10, and TP53* [48,49]), were not significant in our cohort and, therefore, not included in our model. Moreover, the lack of consensus on a standardized methodology, in particular the missing agreement on the optimal estimation of volume-based 18FDG-PET/CT parameters, affects the interpretation and reproducibility of data. Nevertheless, the population of the clinical trial SAKK38/07 received a homogeneous treatment with R-CHOP14, and the functional PET/CT parameters in this study were centrally generated using a standard procedure.

Another potential weakness of the current study may be the relatively small number of genes included in the mutational analysis panel. However, the gene panel used in this study covered all the mutations nowadays considered to be relevant for the molecular categorization of DLBCL [50], with the sole exception of five genes (*ID3, BCOR, CCND3, NFKBIE*, and *ETV6*). In general, the latter genes are not crucial for the definition of the currently recognized molecular subsets: *ID3* and *BCOR* identify the very small “N1 subgroup” of DLBCL (1–2% of all cases).

Even though further analyses, aiming at evaluating a wider gene panel in other cohorts of homogeneously treated DLBCL patients, will be needed to validate or possibly refine our prognostic algorithm, our results support the hypothesis that our prognostic capacity can be improved by the integration of functional imaging with mutational profiling by high-throughput sequencing. Notably, the association of *SOCS1* mutations (the “ST2 subgroup”) with a favorable outcome was reported in all large-scale genetic studies of DLBCL (five-year OS 84% in the NCI study, 75% in the Harvard study, and 80% in the HMRN study, respectively), which corroborates the findings of our study [50].

In conclusion, the current analysis proposed a prognostic model that, using information available at baseline, predicts response to R-CHOP14 treatment in DLBCL patients. In our prior study showing the prognostic impact of MTV and MH in the SAKK38/07 patient cohort, we were able to validate the results in an independent cohort of patients treated with the more conventional R-CHOP21 regimen [24]. Such validation was not possible for the present model, but there is no biological reason to expect that the prognostic impact of the mutational profile is restricted only to the R-CHOP14-treated patients.

## 5. Conclusions

Our results provide proof of principle that integration of functional PET parameters with mutational profiling can improve risk stratification of DLBCL patients, offering a promising tool for the design of clinical trials focused on treatment optimization for newly diagnosed patients. An international process is ongoing to standardize the estimation of volumetric PET/CT parameters [43,51,52,53] and will hopefully allow a wider use and validation of powerful prognostic models based on integrative PET.

## Figures and Tables

**Table 1:** Main features at baseline in the whole cohort of 141 patients.

Characteristics		Number	%
Sex, male		73	51.8
Age ≥ 60 years		68	48.2
LDH elevated		68	48.2
Extranodal sites > 1		34	24.1
ECOG PS ≥ 2		10	7.1
Stage III-IV		80	56.7
High-intermediate or high-risk IPI		44	31.2
High-risk R-IPI		44	31.2
High-intermediate or high-risk NCCN-IPI		52	36.9
Germinal center B-like subtype (COO tested in 113)		84	74.3
cMYC and BCL-2 double expression (tested in 87)		12	13.8
MTV	≥931 mL (cut-off point for PFS)	46 34	32.6 24.1
	≥1149 mL (cut-off point for OS)		
MH ≥ 0.43 AUC-CSH		55	39.0

Abbreviations: LDH, lactate dehydrogenase; ECOG PS, performance status according to Eastern Cooperative Oncology Group criteria; IPI, International Prognostic Index; R-IPI, revised IPI; NCCN-IPI, National Comprehensive Cancer Network enhanced IPI; COO, cell of origin; MTV, metabolic tumor volume; OS, overall survival; PFS, progression-free survival; MH, metabolic heterogeneity; AUC-CSH: area under the curve of cumulative SUV histogram.

**Table 2:** Impact on progression-free survival of the mutations found in 46 of the assessed genes, in which mutations were found.

Mutated Gene	Frequency N = 72 (100%)	No Progression or Death N = 56 (77.8%)	Progression or Death N = 16 (22.2%)	*p*-Value
*ATM*	14 (19.4%)	11 (19.6%)	3 (18.8%)	0.937
*B2M*	11 (15.3%)	9 (16.1%)	2 (12.5%)	0.726
*BCL2*	4 (3.6%)	3 (5.4%)	1 (6.2%)	0.891
*BCL6*	2 (2.8%)	2 (3.6%)	0 (0%)	0.443
*BCL10*	3 (4.2%)	3 (5.4%)	0 (0%)	0.344
*BTG1*	8 (11.1%)	8 (14.3%)	0 (0%)	0.109
*CARD11*	9 (12.5%)	5 (8.9%)	4 (25.0%)	0.086
*CD79B*	4 (3.6%)	2 (3.6%)	2 (12.5%)	0.169
*CREBBP_EP300*	14 (19.4%)	7 (12.5%)	7 (43.8%)	0.005
*EBF1*	3 (4.2%)	3 (5.4%)	0 (0%)	0.344
*EZH2*	10 (13.9%)	8 (14.3%)	2 (12.5%)	0.855
*FOXO1*	3 (4.2%)	3 (5.4%)	0 (0%)	0.344
*GNA13*	13 (18.1%)	10 (17.9%)	3 (18.8%)	0.935
*HIST1H1C*	7 (9.7%)	6 (10.7%)	1 (6.2%)	0.595
*IDH1*	1 (1.4%)	1 (1.8%)	0 (0%)	0.590
*IKZF1*	2 (2.8%)	2 (3.6%)	0 (0%)	0.443
*IRF4*	1 (1.4%)	1 (1.8%)	0 (0%)	0.590
*JAK2*	1 (1.4%)	1 (1.8%)	0 (0%)	0.590
*KLHL6*	1 (1.4%)	1 (1.8%)	0 (0%)	0.590
*KMT2D*	25 (34.7%)	20 (35.7%)	5 (31.2%)	0.741
*KMT2C*	2 (2.8%)	2 (3.6%)	0 (0%)	0.443
*KRAS*	1 (1.4%)	1 (1.8%)	0 (0%)	0.590
*MCL1*	3 (4.2%)	3 (5.4%)	0 (0%)	0.344
*MEF2B*	6 (8.3%)	5 (8.9%)	1 (6.2%)	0.732
*MYC*	5 (6.9%)	4 (7.1%)	1 (6.2%)	0.901
*MYD88*	5 (6.9%)	3 (5.4%)	2 (12.5%)	0.322
*NOTCH1*	1 (1.4%)	1 (1.8%)	0 (0%)	0.590
*NOTCH2*	3 (4.2%)	2 (3.6%)	1 (6.2%)	0.636
*PAX5*	2 (2.8%)	2 (3.6%)	0 (0%)	0.443
*PIK3CD*	2 (2.8%)	2 (3.6%)	0 (0%)	0.443
*PIK3R1*	1 (1.4%)	1 (1.8%)	0 (0%)	0.590
*PIM1*	7 (9.7%)	6 (10.7%)	1 (6.2%)	0.595
*PRDM1*	2 (2.8%)	1 (1.8%)	1 (6.2%)	0.338
*PTEN*	5 (6.9%)	4 (7.1%)	1 (6.2%)	0.901
*PTPN1*	2 (2.8%)	2 (3.6%)	0 (0%)	0.443
*RELN*	1 (1.4%)	1 (1.8%)	0 (0%)	0.590
*RHOA*	1 (1.4%)	1 (1.8%)	0 (0%)	0.590
*SGK1*	4 (3.6%)	3 (5.4%)	1 (6.2%)	0.891
*SOCS1*	19 (26.4%)	18 (32.1%)	1 (6.2%)	0.038
*STAT6*	6 (8.3%)	5 (8.9%)	1 (6.2%)	0.732
*TET2*	3 (4.2%)	1 (1.8%)	2 (12.5%)	0.122
*TNFAIP3*	11 (15.3%)	10 (17.9%)	1 (6.2%)	0.255
*TP53*	8 (11.1%)	6 (10.7%)	2 (12.5%)	0.841
*U2AF1*	1 (1.4%)	1 (1.8%)	0 (0%)	0.590
*XPO1*	2 (2.8%)	2 (3.6%)	0 (0%)	0.443

**Table 3:** Patient features according to the prognostic group defined by the classification tree for PFS.

Characteristics	Low Risk (N = 12)	Intermediate Risk (N = 108)	High Risk (N = 21)	*p*-Value
	*n* (%)	*n* (%)	*n* (%)	
Age				0.061
*≥60 years*	2 (16.7)	54 (50.0)	12 (57.1)	
LDH				<0.001
*Elevated*	8 (66.7)	42 (38.9)	18 (85.7)	
Extranodal sites				0.159
>1	1 (8.3)	25 (23.1)	8 (38.7)	
ECOG PS				0.269
0–1	12 (100.0)	101 (93.5)	18 (85.7)	
Ann Arbor stage				0.019
*I-II*	8 (66.7)	49 (45.4)	4 (19.0)	
*III-IV*	4 (33.3)	59 (54.6)	17 (81.0)	
IPI risk group		54 (50.0)		0.012
*Low risk*	8 (66.7)	23 (21.3)	3 (14.3)	
*Low-intermediate risk*	3 (25.0)	20 (18.5)	6 (28.6)	
*High-intermediate risk*	0 (0.0)	11 (10.2)	7 (33.3)	
*High risk*	1 (8.3)		5 (23.8)	
COO (n = 113)				0.669
*GCB*	4 (33.3)	22 (25.9)	3 (18.8)	
*non-GCB*	8 (66.7)	63 (74.1)	13 (81.2)	
Mutational profile (n = 72)				<0.001
*Favorable (SOCS1mut and CREBBP/EP300wt)*	12 (100.0)	3 (5.9)	1 (11.1)	
*Unfavorable (SOCS1wt and/or CREBBP/EP300mut)*	0	48 (94.1)	8 (88.9)	

Abbreviations: N, number; LDH, lactate dehydrogenase; ECOG PS, performance status according to Eastern Cooperative Oncology Group criteria; IPI, International Prognostic Index; COO, cell of origin; GCB, germinal center B.

**Table 4:** Cox regression models for either PFS or OS prediction including the International Prognostic Indices and the integrative PET model derived from the classification tree.

*Prognostic Indices*	*PFS*			*OS*		
	*HR*	95% *CI*	*p-*Value	*HR*	95% *CI*	*p-*Value
PET/mutational Model	4.42	2.07 to 9.45	<0.001	5.96	2.41 to 14.73	<0.001
IPI	0.93	0.47 to 1.85	0.839	0.75	0.34 to 1.64	0.470
R-IPI	1.33	0.43 to 4.13	0.623	1.54	0.35 to 6.73	0.563
NCCN_IPI	1.04	0.54 to 1.99	0.906	1.56	0.70 to 3.48	0.278

Abbreviations: PFS, progression-free survival; OS, overall survival; HR, hazard ratio; 95% CI, 95% confidence interval; IPI, International Prognostic Index; R-IPI, revised IPI; NCCN-IPI, National Comprehensive Cancer Network enhanced IPI.

**Table 5:** Comparison of international indices with integrative PET model derived from the classification tree.

*Prognostic Indices*		*PFS*		*OS*
	*AIC*	*CPE*	*AIC*	*CPE*
PET/mutational Model	257	0.67	199	0.69
IPI	273	0.59	215	0.61
R-IPI	272	0.59	214	0.62
NCCN-IPI	272	0.58	211	0.64

Abbreviations: PFS, progression-free survival; OS, overall survival; AIC, Akaike information criterion; CPE, concordance probability estimate; IPI, International Prognostic Index; R-IPI, revised IPI; NCCN-IPI, National Comprehensive Cancer Network enhanced IPI.

## Data Availability

The datasets generated and/or analyzed during the current study are not publicly available due to legal restrictions. De-identified individual data used for this analysis are currently being shared with the PETRA (PET re-analysis; last access: 1 January 2021, https://www.petralymphoma.org) consortium. Further data sharing could be possible on a reasonable request addressed to the SAKK—Swiss Group for Clinical Cancer Research (https://www.sakk.ch/en/contact, accessed on 1 January 2021).
